# Effect of Integrating Machine Learning Mortality Estimates With Behavioral Nudges to Clinicians on Serious Illness Conversations Among Patients With Cancer: A Stepped-Wedge Cluster Randomized Clinical Trial

**DOI:** 10.1001/jamaoncol.2020.4759

**Published:** 2020-10-15

**Authors:** Christopher R. Manz, Ravi B. Parikh, Dylan S. Small, Chalanda N. Evans, Corey Chivers, Susan H. Regli, C. William Hanson, Justin E. Bekelman, Charles A. L. Rareshide, Nina O’Connor, Lynn M. Schuchter, Lawrence N. Shulman, Mitesh S. Patel

**Affiliations:** 1 Department of Population Sciences, Dana-Farber Cancer Institute, Boston, Massachusetts; 2 Department of Medicine, Perelman School of Medicine, University of Pennsylvania, Philadelphia; 3 Penn Center for Cancer Care Innovation, Abramson Cancer Center, University of Pennsylvania, Philadelphia; 4 Corporal Michael J. Crescenz Veterans Affairs Medical Center, Philadelphia, Pennsylvania; 5 Wharton School of the University of Pennsylvania, Philadelphia; 6 Penn Medicine Nudge Unit, Philadelphia, Pennsylvania; 7 University of Pennsylvania Health System, Philadelphia

## Abstract

**Importance:**

Serious illness conversations (SICs) are structured conversations between clinicians and patients about prognosis, treatment goals, and end-of-life preferences. Interventions that increase the rate of SICs between oncology clinicians and patients may improve goal-concordant care and patient outcomes.

**Objective:**

To determine the effect of a clinician-directed intervention integrating machine learning mortality predictions with behavioral nudges on motivating clinician-patient SICs.

**Design, Setting, and Participants:**

This stepped-wedge cluster randomized clinical trial was conducted across 20 weeks (from June 17 to November 1, 2019) at 9 medical oncology clinics (8 subspecialty oncology and 1 general oncology clinics) within a large academic health system in Pennsylvania. Clinicians at the 2 smallest subspecialty clinics were grouped together, resulting in 8 clinic groups randomly assigned to the 4 intervention wedge periods. Included participants in the intention-to-treat analyses were 78 oncology clinicians who received SIC training and their patients (N = 14 607) who had an outpatient oncology encounter during the study period.

**Interventions:**

(1) Weekly emails to oncology clinicians with SIC performance feedback and peer comparisons; (2) a list of up to 6 high-risk patients (≥10% predicted risk of 180-day mortality) scheduled for the next week, estimated using a validated machine learning algorithm; and (3) opt-out text message prompts to clinicians on the patient’s appointment day to consider an SIC. Clinicians in the control group received usual care consisting of weekly emails with cumulative SIC performance.

**Main Outcomes and Measures:**

Percentage of patient encounters with an SIC in the intervention group vs the usual care (control) group.

**Results:**

The sample consisted of 78 clinicians and 14 607 patients. The mean (SD) age of patients was 61.9 (14.2) years, 53.7% were female, and 70.4% were White. For all encounters, SICs were conducted among 1.3% in the control group and 4.6% in the intervention group, a significant difference (adjusted difference in percentage points, 3.3; 95% CI, 2.3-4.5; *P* < .001). Among 4124 high-risk patient encounters, SICs were conducted among 3.6% in the control group and 15.2% in the intervention group, a significant difference (adjusted difference in percentage points, 11.6; 95% CI, 8.2-12.5; *P* < .001).

**Conclusions and Relevance:**

In this stepped-wedge cluster randomized clinical trial, an intervention that delivered machine learning mortality predictions with behavioral nudges to oncology clinicians significantly increased the rate of SICs among all patients and among patients with high mortality risk who were targeted by the intervention. Behavioral nudges combined with machine learning mortality predictions can positively influence clinician behavior and may be applied more broadly to improve care near the end of life.

**Trial Registration:**

ClinicalTrials.gov Identifier: NCT03984773

## Key Points

**Question:**

What is the effect of delivering machine learning mortality predictions with behavioral nudges to oncology clinicians on the rate of serious illness conversations with patients with cancer?

**Findings:**

In this stepped-wedge cluster randomized clinical trial that included 14 607 patients with cancer, the intervention led to a significant increase in serious illness conversations from approximately 1% to 5% of all patient encounters and from approximately 4% to 15% of encounters with patients having high predicted mortality risk.

**Meaning:**

Machine learning mortality predictions combined with behavioral nudges to clinicians led to an increased rate of serious illness conversations for patients with cancer.

## Teaser

This stepped-wedge cluster randomized clinical trial assesses the effect of a clinician-directed intervention combining machine learning mortality predictions with behavioral nudges vs usual care on motivating serious illness conversations between clinicians and patients with cancer.

## Introduction

Patients with cancer often receive treatment and acute care use, particularly near the end of life, that does not align with their preferences or goals of care.^1,2,3,4,5^ Early discussions about goals and treatment preferences may lead to better perceived quality of life, reduced emotional distress, and decreased health care use near the end of life.^6,7,8^ However, most patients with cancer die without a documented discussion about goals and treatment preferences.^9,10^

There are several reasons for the dearth of such discussions between oncologists and patients with cancer. First, oncologists correctly identify fewer than half of their patients who will die within 6 to 12 months.^11,12^ Overoptimistic assessments of short-term mortality risk may result in inadequate identification of patients who will benefit from a timely discussion.^13,14,15^ Second, it is often difficult to change clinician behavior and practice patterns. Insights from behavioral economics, including changing default options and delivering performance feedback, have been shown to alter clinician behavior in a variety of clinical settings.^16,17,18,19^

Serious illness conversations (SICs) are a type of advance care planning discussion and are structured conversations between clinicians, patients, and families intended to explore prognostic awareness, treatment priorities, and goals of care.^20^ In a prior randomized clinical trial, an intervention that provided clinicians with a Serious Illness Conversation Guide ameliorated anxiety and depression among patients with cancer.^21,22^ The objective of the present stepped-wedge cluster randomized clinical trial was to test the effect of an intervention that integrated real-time machine learning (ML) mortality predictions with behavioral nudges on motivating SICs between oncologists and high-risk patients. We used a validated ML algorithm that has been shown to accurately predict 180-day mortality risk.^23^ We hypothesized that this multimodal intervention would increase the rate of SICs between oncology clinicians and all patients as well as between oncology clinicians and high-risk patients.

## Methods

### Study Design

This was a stepped-wedge cluster randomized clinical trial (NCT03984773) among 9 medical oncology clinics in the University of Pennsylvania Health System.^24^ The trial included five 4-week wedges and was conducted from June 17 to November 1, 2019. The trial evaluated the effect of delivering ML mortality estimates with behavioral nudges on the rate of SICs among patients with cancer. The mortality prediction algorithm has been previously validated.^23^ This trial followed the Consolidated Standards of Reporting Trials (CONSORT) reporting guideline. The trial protocol was approved by the University of Pennsylvania institutional review board (trial protocol in Supplement 1),^25^ which also granted a waiver for the requirement to obtain informed consent because this study was an evaluation of a health system initiative that posed minimal risk to clinicians and patients.

### Study Sample

Eligible clinicians were physicians, physician assistants, and nurse practitioners who provided oncology care at 8 subspecialty clinics (breast, central nervous system, gastrointestinal, genitourinary, lymphoma, melanoma, myeloma, and thoracic or head and neck) and 1 general oncology clinic (n = 78). Clinicians at the 2 smallest subspecialty clinics (melanoma and central nervous system malignant neoplasms) were grouped together, resulting in 8 clinic groups. Clinicians were excluded if they cared only for patients with benign hematologic or genetic disorders (n = 3) saw fewer than 12 patients classified as high risk by the algorithm in either the preintervention or postintervention period (n = 6), or had not undergone SIC training at the time of trial initiation (n = 4).

Eligible patients had encounters at 1 of the clinics during the study period. Patients were excluded if they had a documented SIC or advanced care planning conversation (ACP) prior to the start of the trial, or if they were enrolled in another ongoing trial of early palliative care. Genetics encounters were also excluded.

### Randomization

The 8 clinic groups were randomized to the 4 intervention wedge periods, stratified by SIC rate (above or below the median). The study senior author (M.S.P.) and data analyst (C.A.L.R.) were blinded to wedge assignment until analysis of the primary end point was complete. Blinding was not feasible for investigators who were oncologists practicing at Penn Medicine.

### Interventions

All clinicians included in the trial were trained in the use of the Serious Illness Conversation Guide (Ariadne Labs) at least 3 months prior to the start of the trial (range, 5-18 months).^20^ The default advance care planning template for clinicians was based on this guide.

For approximately 1 year before the trial began, clinicians in all clinic groups received weekly emails that reported their individual SIC performance and blinded peer comparison in 2 bar graphs: 1 for cumulative SICs performed since that clinician’s SIC training date and 1 for SICs performed in the prior week. This continued during the control period of the trial and was turned off once the intervention was implemented. In the first 4-week wedge, all clinic groups remained in usual care (control). In each subsequent 4-week period, 2 clinic groups (intervention) received the intervention. By the start of the fifth wedge, all clinic groups were receiving the intervention (eFigure 1 in the Supplement 2).

The intervention was directed toward clinicians and included 3 main components. First, every Thursday, each clinician received an email with performance feedback indicating the number of SICs he or she performed in the prior 4 weeks. The email also contained a peer comparison message; this message differed for clinicians who performed below or above 8 SICs or who were among the top 10 performers of SICs during the prior 4 weeks. Samples of the email text are shown in eFigure 2 in the Supplement 2. Performance feedback and peer comparisons have been shown to be successful in motivating clinician decision-making.^17,26,27^

Second, the email included a link to a secure dashboard in which clinicians could review patients scheduled for the following week in their clinic who had a high risk of mortality; these were described as patients “who may benefit from an SIC.” Mortality risk was determined by a previously validated ML algorithm, which used structured electronic health record (EHR) data to predict risk of 180-day mortality.^2^ Every Thursday morning, the algorithm generated mortality risk predictions for all patients with a scheduled encounter during the following week with an eligible clinician. Clinicians could view a personalized list of up to 6 patients with the highest predicted 180-day mortality risk (“high-risk” patients) via a secure dashboard. Patients with a mortality risk below 10% were not included in the list. Mortality predictions were updated weekly; thus, patient assignment as “high risk” could change from encounter to encounter. Information in the dashboard included patient identifiers, the author and date of any prior SICs, and a checkbox to opt out of reminder texts. Using opt out (as opposed to opt in) as a default has been successful in different health care settings to change physician behavior.^28,29,30,31,32^ The prediction algorithm silently identified high-risk patients during the control periods, but these lists were not shared with the clinicians via email or text.

Third, clinicians received a text message reminding them to consider having an SIC with any high-risk patient. For the high-risk patients on the clinicians’ dashboard, clinicians were sent this message on the morning of the patient’s encounter, unless they opted out or an SIC was performed in the prior 2 months.

### Outcome Measures

The primary outcome was change in SIC rate among all patient encounters. Secondary outcomes included changes in rates of (1) SICs among high-risk patients and (2) ACPs for the overall sample and for the high-risk subgroup.

The electronic medical record had an ACP section in which advance care planning notes were documented. Any note documented in this section was classified as an ACP. The subset of notes that included a preformatted SIC template was classified as an SIC. Thus, ACPs encompassed both SIC and non-SIC documentation about treatment goals and wishes.

### Statistical Analysis

A priori power calculations used data from the University of Pennsylvania Health System to estimate the rate of SICs. Assuming a baseline SIC rate of 0.65%, we estimated that this trial would have 80% power to detect a 60% relative increase in SIC rate using a 2-sided α of 0.05 as our threshold for statistical significance.

All trial clinicians and their patients were included in the intention-to-treat analysis. Analyses were conducted using the first patient encounter in each wedge; subsequent encounters were excluded to avoid biasing results toward patients with numerous encounters. Patients seen by physician assistants and nurse practitioners were allocated to their corresponding oncologist. Because care planning documentation can occur days to weeks after an encounter, SICs and ACPs within 28 days of the encounter with no intervening encounter were counted in the primary outcome. We performed a sensitivity analysis that defined the primary outcome using a shorter, 7-day period after the index encounter to count SICs and ACPs.

Similar to prior work,^7^ we fit models for the outcome measures based on generalized estimating equations with a logit link and an independence correlation structure using oncologist as the clustering unit. The main models used patient-encounter level observations, clinic-group and wedge-period fixed effects, and a binary indicator for intervention implementation. To test the robustness of our findings, we fit a fully adjusted model that also included age, sex, marital status, patient-reported race/ethnicity, insurance status, and Charlson Comorbidity Index. To obtain the adjusted difference and 95% CIs in the percentage points, we used the bootstrap method, resampling patients 1000 times.^33,34^ Resampling of patients was conducted by the oncologist variable to maintain clustering at the oncologist level. Analyses were conducted between January 6 and March 9, 2020. Two-sided hypothesis tests used a significance level of *P* ≤ .05. All analyses were conducted using SAS, version 9.4 (SAS Institute Inc).

## Results

The sample consisted of 78 clinicians (of whom 42 were oncologists), 14 607 patients, and 26 059 patient encounters (Figure). Patients’ mean (SD) age was 61.9 (14.2) years. Of 14 607 patients, 8141 (55.7%) were female, 10 285 (70.4%) were non-Hispanic White, 6876 (47.1%) were commercially insured, 6806 (46.7%) were covered by Medicare, and 925 (6.3)% were covered by Medicaid (Table 1). Patients in the control and intervention groups had similar characteristics. High-risk encounters accounted for 2125 of 12 170 patient encounters (17.5%) in the control groups and 1999 of 13 889 patient encounters (14.4%) in the intervention groups. The median 180-day mortality risk per patient-encounter was similar in the control and intervention groups (4% [interquartile range {IQR}, 2%-13%] vs 4% [IQR, 2%-12%]). Among high-risk patients, the patients’ characteristics and median mortality risk (34% [IQR, 20%-53%] vs 36% [IQR, 20%-56%]) were also similar between control and intervention groups (eTable 1 in Supplement 2). The number of patient encounters and SIC rates by clinic and control vs intervention group are shown in eTable 2 in Supplement 2.

**Figure.**
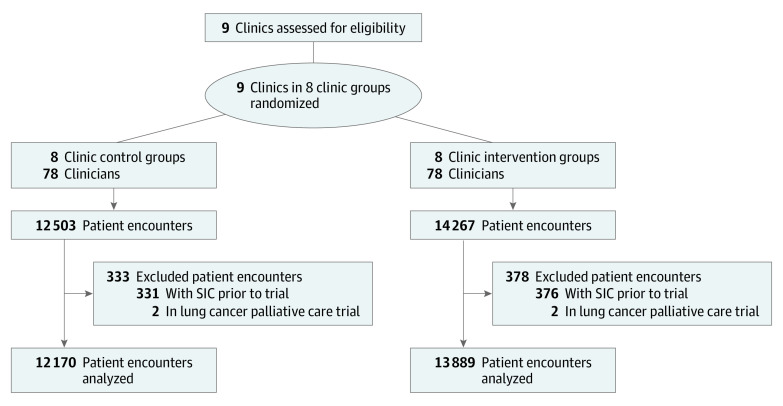
CONSORT Diagram SIC indicates serious illness conversation.

**Table 1.** Patient-Encounter Characteristics

Characteristic	No. (%) of patient encounters		*P* value
	Control (n = 12 170)	Intervention (n = 13 889)	
Sociodemographic characteristics			
Age, mean (SD), y	62.5 (13.8)	61.3 (14.5)	<.001
Female	6426 (52.8)	7576 (54.5)	.005
Race/ethnicity			
Non-Hispanic White	8629 (70.9)	9709 (69.9)	.02
Non-Hispanic Black	2436 (20.0)	2772 (20.0)	
Other	1105 (9.1)	1408 (10.1)	
Insurance			
Commercial	5467 (44.9)	6522 (47.0)	<.001
Medicare	5953 (48.9)	6321 (45.5)	
Medicaid	750 (6.2)	1046 (7.5)	
Clinical imblances^a^			
Breast	2003 (16.5)	1520 (10.9)	<.001
Central nervous system + melanoma	762 (6.3)	1188 (8.6)	
Gastrointestinal	624 (5.1)	2770 (19.9)	
General oncology	2171 (17.8)	3757 (27.1)	
Genitourinary	1792 (14.7)	451 (3.2)	
Myeloma	2592 (21.3)	707 (5.1)	
Lymphoma	541 (4.4)	2289 (16.5)	
Thoracic	1685 (13.8)	1207 (8.7)	
Clinical characteristics			
Charlson Comorbidity Index, median (IQR)	3 (2-8)	3 (2-8)	.02
Predicted mortality risk, median (IQR), %^b^	4 (2-13)	4 (2-12)	<.001
High-risk patient encounters (% of total)	2125 (17.5)	1999 (14.4)	<.001

Abbreviation: IQR, interquartile range.

^a^ Imbalances between control and intervention groups were due to staggered intervention start times in the stepped-wedge design.

^b^ Mortality risk at 180 days as predicted by the mortality prediction algorithm.

### Serious Illness Conversations

Among all patients, SICs were conducted in 155 of 12 170 control encounters (1.3%) and 632 of 13 889 intervention encounters (4.6%). Among high-risk patients, SICs were conducted in 77 of 2125 control encounters (3.6%) and 304 of 1999 intervention encounters (15.2%) (eFigure 3 in Supplement 2). In adjusted analyses of all patients, the intervention led to a significant increase in the SIC rate (adjusted difference in percentage points, 3.3; 95% CI, 2.3-4.5; *P* < .001) (Table 2). In adjusted analyses of 4124 high-risk patient encounters, there was a significant increase in the SIC rate (adjusted difference in percentage points, 11.6; 95% CI, 8.2-12.5; *P* < .001). Full model results are shown in eTable 3 and eTable 4 in Supplement 2. Sensitivity analyses showed similar results (eTables 5, 6, 7, and 12 in Supplement 2).

**Table 2.** Adjusted Changes in Serious Illness Conversations and in Advanced Care Planning

Conversation	No./total No. (%) of encounters		Adjusted difference for intervention relative to control, percentage points (95% CI)^a^	*P* value
	Control	Intervention		
Serious illness encounters				
All patients	155/12 170 (1.3)	632/13 889 (4.6)	3.3 (2.3-4.5)	<.001
High-risk patients	77/2125 (3.6)	304/1999 (15.2)	11.6 (8.2-15.5)	<.001
Advanced care planning encounters				
All patients	231/12 170 (1.9)	680/13 889 (4.9)	3.0 (2.1-4.1)	.001
High-risk patients	124/2125 (5.8)	350/1999 (17.5)	11.7 (8.4-15.7)	<.001

^a^ Adjusted for intervention, clinic group, and wedge.

### Advanced Care Planning Conversations

Among all patients, ACPs were conducted in 231 of 12 170 control encounters (1.9%) and 680 of 13 889 intervention encounters (4.9%). Among high-risk patients, ACPs were conducted in 124 of 2125 control encounters (5.8%) and 350 of 1999 intervention encounters (17.5%). In adjusted analyses of all patients, the intervention led to a significant increase in the ACP rate (adjusted difference in percentage points, 3.0; 95% CI, 2.1-4.1; *P* = .001) (Table 2). In adjusted analyses of high-risk patients, the intervention led to a significant increase in the ACP rate (adjusted difference in percentage points, 11.7; 95% CI, 8.4-15.7; *P* < .001). Full model results are shown in eTable 8 and eTable 9 in Supplement 2. Sensitivity analyses showed similar results (eTables 10, 11 and 12 in Supplement 2). No adverse events were reported.

## Discussion

In this randomized clinical trial conducted in 9 oncology practices, an intervention that combined ML mortality predictions with behavioral nudges significantly increased rates of SICs and ACPs. The intervention led to a 4-fold increase (3.6%-15.2%) in SICs among high-risk patients targeted by the intervention. To our knowledge, this is one of the first randomized clinical trials testing the effect of real-time ML predictions and behavioral nudges on clinical care. There are several important implications of our findings.

First, our intervention resulted in a large increase in SIC rates, especially among patients at highest risk of 180-day mortality. Although prior studies have assessed SIC and ACP rates among decedents,^6,7^ our study showed that a behaviorally informed intervention led to a rapid increase in such conversations among all patients with cancer. Although the optimal SIC prevalence among patients with cancer is unknown, the effect of this intervention compares favorably with other published interventions on increasing the number of such conversations.^35,36,37^ Discussions about goals and treatment preferences have been associated with less aggressive care for cancer patients near the end of life, including increased hospice utilization rates and reduced chemotherapy and hospitalization rates near the end of life.^1,2,3,4,5,6,7,8^ Furthermore, documentation of such discussions ensures that patients’ wishes are recorded and available across care settings in a health system. Our study has particular importance as oncologists and patients now grapple with care delays and competing risks due to the coronavirus disease 2019 pandemic. Indeed, documentation of goals and wishes has been recognized by several oncology guideline bodies as a key priority to ensure goal-concordant care during the pandemic. A uniform approach to the automated identification of high-risk patients that increases the rate of SICs can be combined with new care models, such as telemedicine, to meet this need.

Targeting SICs to high-risk patients at risk of short-term mortality may result in improved goal-concordant care, which was not shown in a previous randomized clinical trial of an intervention consisting primarily of training on the Serious Illness Conversation Guide.^21,22^ The cohort in that trial consisted of individuals who died. The use of that cohort may explain why overall rates of SICs in the control and intervention groups were much higher than those in the present trial, which consisted of all patients (decedents and nondecedents) with cancer, including many with curable early-stage cancers for whom SICs would not be expected. In addition, the relatively low SIC prevalence among even high-risk patients after the intervention may be due to the exclusion of patients with an existing SIC at the start of the present trial, a short follow-up period, clinician time constraints, the performance of SICs that were not documented in a trackable fashion in the EHR, and misclassification of patients as high risk. Subsequent research should determine longer-term clinical and quality-of-life outcomes associated with SICs prompted by this intervention.

Second, although several trials have prospectively integrated ML risk predictions into clinical care, few studies have shown that interventions that involve ML predictions can positively influence clinical care or decision-making.^38,39^ Our intervention illustrates that generalizable behavioral principles combined with ML predictions may inform more accurate, individualized prognoses that clinicians and patients use to guide treatment decisions and ensure receipt of goal-concordant care. A key innovation of the present study was integrating behavioral economic principles into the ML-based intervention, including peer comparison, performance feedback, and opt-out reminders, which have been used successfully in other clinical settings to change clinician behavior.^16,17,18,19,40^ Future implementation of artificial intelligence or ML tools in clinical practice should explore similar mechanisms of delivering predictions and integrating behavioral principles to maximally improve clinician and patient decision-making. Yet these behavioral nudges may not be equally necessary and effective. Although the present study cannot disentangle the effects of individual intervention components, setting the default to a desired behavior—in this instance, defaulting clinicians to receive notifications encouraging SICs with high-risk patients—is a powerful nudge that has been widely applied in medicine to change behavior and may be the most impactful component.^28^ Future research should use head-to-head comparisons to identify which intervention components deliver the most benefit and how an integrated EHR-based mortality prediction tool can be generalized to other health systems.

Third, although our intervention effect was strongest in the high-risk patients flagged by the algorithm, we still detected an increase of nearly 2 percentage points in SICs among patients who were not deemed high-risk by the algorithm. These “off-target” patients comprised the majority of our cohort. To our knowledge, off-target effects of an intervention to promote supportive care have not been shown. This off-target effect may have occurred because the clinicians who received the intervention became accustomed to actively considering mortality risk in their clinical practice, resulting in an increase in SICs for patients who may have a poor prognosis but were not flagged by the algorithm as high risk.

### Strengths and Limitations

Our study has several strengths, including the application of behavioral insights into an ML-based intervention, integration of accurate individual-level mortality risk predictions at the point of care, a stepped-wedge design that controlled for cancer site–specific uptake of SICs, and a large sample of patients across several different cancer types and in both tertiary academic and general oncology practices. This study has several limitations. First, this study was performed in a single academic health system, and the participants may not be representative of the general population of oncologists or patients with cancer. However, given the prominence of EHRs in oncology care and the many examples of EHR-based ML algorithms across EHR types, the principles behind this intervention could theoretically be applied in any cancer care setting.^41^ Second, early ACPs and SICs are surrogates for goal-concordant care and decreased aggressive end-of-life care. Longer-term studies are needed to assess whether algorithm-based interventions improve clinical outcomes for patients. Third, our intervention consisted of several components, including a real-time prediction algorithm, a secure list of high-risk patients, peer comparisons, performance reports, and text messages. Future work is needed to assess the individual effects of each of these interventions.

## Conclusions

An intervention combining ML mortality risk predictions and behavioral nudges led to an increase in SICs among patients with cancer. This study provides guidance for more robust integration of automated risk predictions alongside behavioral interventions in future prospective studies related to and outside of SICs in oncology.

## Notes

Supplement 1. **Trial Protocol**

Supplement 2. **eFigure 1.** Stepped-Wedge Design Overview **eFigure 2.** Example Clinician Emails by Performance **eFigure 3.** Serious Illness Conversations for High-Risk Patients by Clinic **eTable 1.** High-Risk Patient Encounter Characteristics **eTable 2.** Patient Encounters and SIC Rates by Clinic and Control vs Intervention **eTable 3.** Regression Table for Serious Illness Conversation Model Including All Patients **eTable 4.** Regression Table for Serious Illness Conversation Model Including High Risk Patients Only **eTable 5.** Fully Adjusted Models for Serious Illness Conversations (SICs) for All Patients and for High Risk Patients Only **eTable 6.** Serious Illness Conversations (SICs) for All Patients, Documented Within 7 Days **eTable 7.** Serious Illness Conversations (SICs), Adjusted for Clinician Time From SIC Guide Training to Trial Start Date **eTable 8.** Regression Table for Advance Care Planning Model Including All Patients **eTable 9.** Regression Table for Advance Care Planning Model Including High Risk Patients Only **eTable 10.** Fully Adjusted Models for Advance Care Planning (ACPs) for All Patients and for High Risk Patients Only **eTable 11.** Adjusted Change in Advance Care Planning Conversations (SICs), Documented Within 7 days **eTable 12.** Adjusted Change in Serious Illness Conversations (SICs) and Advanced Care Planning for Non-High-Risk Patients

Supplement 3. **Data Sharing Statement**
