# Supplementary material for: Effect of Integrating Machine Learning Mortality Estimates With Behavioral Nudges to Clinicians on Serious Illness Conversations Among Patients With Cancer: A Stepped-Wedge Cluster Randomized Clinical Trial
Source: JAMA Oncol. 2020 Oct 15;6(12):e204759. doi: 10.1001/jamaoncol.2020.4759 (PMC7563672; doi:10.1001/jamaoncol.2020.4759)
Supplement: Supplement 2. — eFigure 1. Stepped-Wedge Design Overview eFigure 2. Example Clinician Emails by Performance eFigure 3. Serious Illness Conversations for High-Risk Patients by Clinic eTable 1. High-Risk Patient Encounter Characteristics eTable 2. Patient Encounters and SIC Rates by Clinic and Control vs Intervention eTable 3. Regression Table for Serious Illness Conversation Model Including All Patients eTable 4. Regression Table for Serious Illness Conversation Model Including High Risk Patients Only eTable 5. Fully Adjusted Models for Serious Illness Conversations (SICs) for All Patients and for High Risk Patients Only eTable 6. Serious Illness Conversations (SICs) for All Patients, Documented Within 7 Days eTable 7. Serious Illness Conversations (SICs), Adjusted for Clinician Time From SIC Guide Training to Trial Start Date eTable 8. Regression Table for Advance Care Planning Model Including All Patients eTable 9. Regression Table for Advance Care Planning Model Including High Risk Patients Only eTable 10. Fully Adjusted Models for Advance Care Planning (ACPs) for All Patients and for High Risk Patients Only eTable 11. Adjusted Change in Advance Care Planning Conversations (SICs), Documented Within 7 days eTable 12. Adjusted Change in Serious Illness Conversations (SICs) and Advanced Care Planning for Non-High-Risk Patients [file jamaoncol-e204759-s002.pdf]

Supplementary Online Content

Manz CR, Parikh RB, Small DS, et al. Effect of integrating machine learning mortality estimates with behavioral nudges to clinicians on serious illness conversations among patients with cancer: a stepped-wedge cluster randomized clinical trial. *JAMA Oncol*. Published online October 15, 2020. doi:10.1001/jamaoncol.2020.4759

eFigure 1. Stepped-Wedge Design Overview
eFigure 2. Example Clinician Emails by Performance
eFigure 3. Serious Illness Conversations for High-Risk Patients by Clinic
eTable 1. High-Risk Patient Encounter Characteristics
eTable 2. Patient Encounters and SIC Rates by Clinic and Control vs Intervention
eTable 3. Regression Table for Serious Illness Conversation Model Including All Patients
eTable 4. Regression Table for Serious Illness Conversation Model Including High Risk Patients Only
eTable 5. Fully Adjusted Models for Serious Illness Conversations (Sics) for All Patients and for High Risk Patients Only
eTable 6. Serious Illness Conversations (Sics) for All Patients, Documented Within 7 Days
eTable 7. Serious Illness Conversations (Sics), Adjusted for Clinician Time From SIC Guide Training to Trial Start Date
eTable 8. Regression Table for Advance Care Planning Model Including All Patients
eTable 9. Regression Table for Advance Care Planning Model Including High Risk Patients Only
eTable 10. Fully Adjusted Models for Advance Care Planning (Acps) for All Patients and for High Risk Patients Only
eTable 11. Adjusted Change in Advance Care Planning Conversations (Sics), Documented Within 7 days
eTable 12. Adjusted Change in Serious Illness Conversations (Sics) and Advanced Care Planning for Non-High-Risk Patients

This supplementary material has been provided by the authors to give readers additional information about their work.

© 2020 Manz CR et al. *JAMA Oncology*

eFigure 1. Stepped-Wedge Design Overview

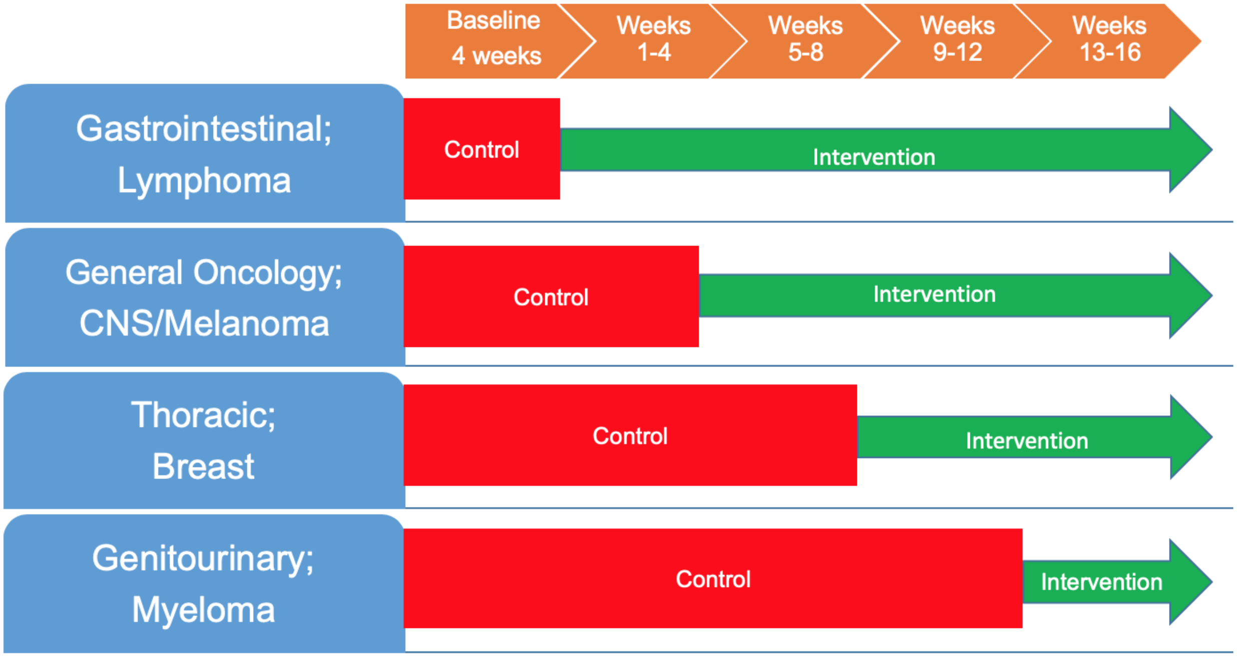

© 2020 Manz CR et al. *JAMA Oncology*

eFigure 2. Example Clinician Emails by Performance

a. Email to clinicians who are not in the top 10 clinicians with the most serious illness conversations in the prior four weeks

Next week's high-risk patients for Serious Illness Conversations

Thursday, July 18, 2019 at 8:05 AM

Show Details

Dear :

The ACC is working to help oncologists have earlier Serious Illness Conversations with patients. In the past four weeks, you have documented **2 conversations.**

18 oncology clinicians have documented more conversations than you during that time.

We have identified patients scheduled to see you next week who may benefit from a Serious Illness Conversation. Click here to view your list (you must be connected to the UPHS network).

Sincerely,

b. Email to the 10 clinicians with the most serious illness conversation in the prior four weeks

Next week's high-risk patients for Serious Illness Conversations

Thursday, July 25, 2019 at 8:05 AM

Show Details

Dear

The ACC is working to help oncologists have earlier Serious Illness Conversations with patients. In the past four weeks, you have documented **11 conversations.**

You are among the top performers - keep up the great work!

We have identified patients scheduled to see you next week who may benefit from a Serious Illness Conversation. Click here to view your list (you must be connected to the UPHS network).

Sincerely,

© 2020 Manz CR et al. *JAMA Oncology*

eFigure 3. Serious Illness Conversations for High-Risk Patients by Clinic

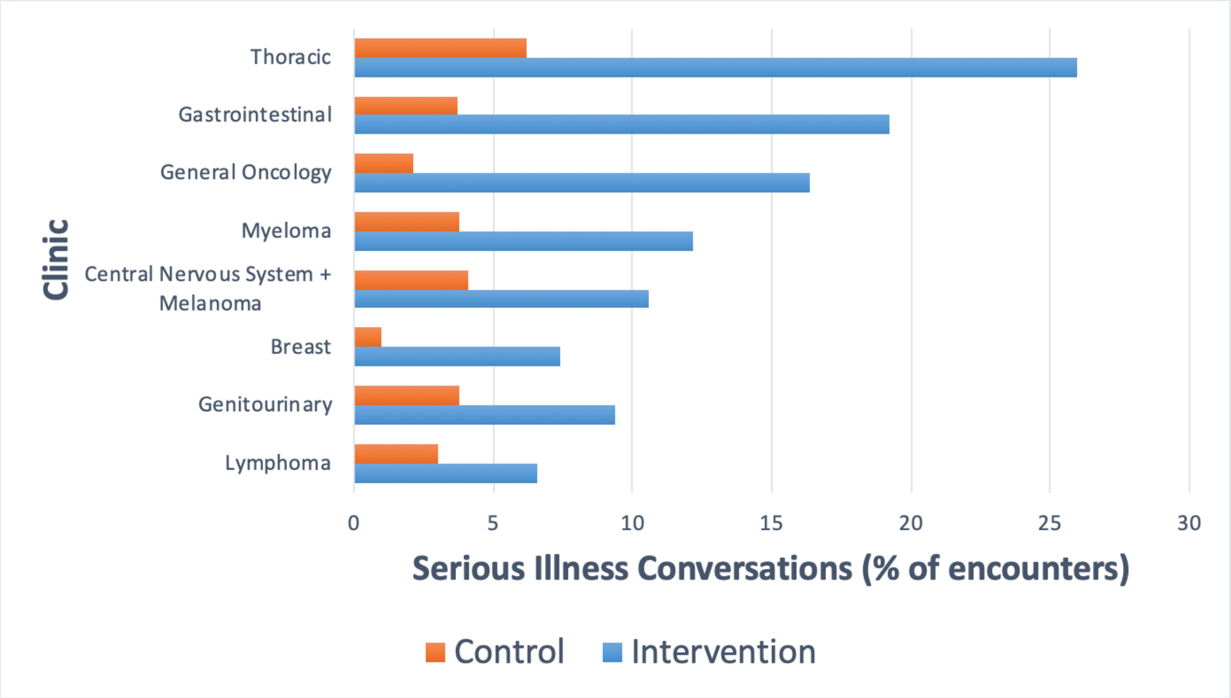

© 2020 Manz CR et al. *JAMA Oncology*

eTable 1. High-Risk Patient Encounter Characteristics

Characteristic	Control (n = 2125)	Intervention (n = 1999)
**Sociodemographics**		
Age, Mean (SD)	64.5 (12.7)	64.4 (13)
Female Gender, No. (%)	1070 (50.4%)	1025 (51.3%)
Race/Ethnicity, No. (%)		
White non-Hispanic	1494 (70.3%)	1417 (70.9%)
Black non-Hispanic	467 (22%)	408 (20.4%)
Other	164 (7.7%)	174 (8.7%)
Insurance, No. (%)		
Commercial	809 (38.1%)	782 (39.1%)
Medicare	1149 (54.1%)	1060 (53%)
Medicaid	167 (7.9%)	157 (7.9%)
**Clinic**		
Breast	311 (14.6%)	204 (10.2%)
Central Nervous System Tumors + Melanoma	145 (6.8%)	161 (8.1%)
Gastrointestinal	136 (6.4%)	527 (26.4%)
General oncology	292 (13.7%)	415 (20.8%)
Genitourinary	318 (15%)	64 (3.2%)
Lymphoma	67 (3.2%)	272 (13.6%)
Myeloma	420 (19.8%)	98 (4.9%)
Thoracic	436 (20.5%)	258 (12.9%)
**Clinical**		
Charlson comorbidity index, median (IQR)	8 (3-9)	8 (3-9)
Predicted mortality risk, mean (SD)*	34% (20-53%)	36% (20-56%)

SD = Standard deviation
IQR = Interquartile range
* 180-day mortality risk as predicted by mortality prediction algorithm

© 2020 Manz CR et al. *JAMA Oncology*

eTable 2. Patient Encounters and SIC Rates by Clinic and Control vs Intervention

	Number of patient encounters		Percent of encounters with SIC (X/Y)	
**Intervention Assignment**	**Control**	**Intervention**	**Control**	**Intervention**
Breast	2003	1520	0.4% (8/2003)	1.6% (25/1520)
Central Nervous System/ Melanoma	762	1188	1.4% (11/762)	4.2% (50/1188)
Gastrointestinal	624	2770	2.7% (17/624)	8.6% (239/2770)
General oncology	2171	3757	1% (21/2171)	3.3% (125/3757)
Genitourinary	1792	451	0.7% (13/1792)	2.2% (10/451)
Lymphoma	541	2289	0.9% (5/541)	1.8% (42/2289)
Myeloma	2592	707	1.2% (32/2592)	2.5% (18/707)
Thoracic	1685	1207	2.8% (48/1685)	10.2% (123/1207)
**Total**	12170	13889	1.3% (155/12170)	4.6% (632/13889)

© 2020 Manz CR et al. *JAMA Oncology*

eTable 3. Regression Table for Serious Illness Conversation Model Including All Patients

Variable	Estimate	P Value	OR (CI)
**Wedge**			
10/7/19 – 11/3/19	0.8552	0.0002	2.35 (1.5-3.69)
7/15/19 – 8/11/19	0.2788	0.1368	1.32 (0.92-1.91)
8/12/19 – 9/8/19	0.5318	0.0074	1.7 (1.15-2.51)
9/9/19 – 10/6/19	0.5809	0.0203	1.79 (1.09-2.92)
**Intervention**			
Pre	Ref	-	Ref
Post	0.7028	<.0001	2.02 (1.44-2.83)
**Clinic**			
Lymphoma	Ref	-	Ref
Gastrointestinal	1.5814	0.0002	4.86 (2.14-11.03)
Thoracic	1.5448	0.0003	4.69 (2.02-10.88)
Central Nervous System / Melanoma	0.7444	0.0664	2.11 (0.95-4.66)
Myeloma	0.2841	0.577	1.33 (0.49-3.61)
Genitourinary	-0.0877	0.8423	0.92 (0.39-2.17)
General oncology	0.4759	0.2153	1.61 (0.76-3.42)
Breast	-0.3874	0.5014	0.68 (0.22-2.1)

© 2020 Manz CR et al. *JAMA Oncology*

eTable 4. Regression Table for Serious Illness Conversation Model Including High Risk Patients Only

Variable	Estimate	P Value	OR (CI)
**Wedge**			
10/7/19 – 11/3/19	1.1016	<.0001	3.01 (1.73-5.22)
7/15/19 – 8/11/19	0.4216	0.0794	1.52 (0.95-2.44)
8/12/19 – 9/8/19	0.637	0.0177	1.89 (1.12-3.2)
9/9/19 – 10/6/19	0.7633	0.009	2.15 (1.21-3.8)
**Intervention**			
Pre	Ref	-	Ref
Post	1.0011	<.0001	2.72 (1.73-4.28)
**Clinic**			
Lymphoma	Ref	-	Ref
Gastrointestinal	1.1453	0.0077	3.14 (1.35-7.29)
Thoracic	1.2996	0.0075	3.67 (1.41-9.52)
Central Nervous System / Melanoma	0.4679	0.3988	1.6 (0.54-4.73)
Myeloma	0.4484	0.3251	1.57 (0.64-3.82)
Genitourinary	0.3504	0.487	1.42 (0.53-3.81)
General oncology	0.7842	0.1208	2.19 (0.81-5.9)
Breast	-0.2734	0.5367	0.76 (0.32-1.81)

© 2020 Manz CR et al. *JAMA Oncology*

eTable 5. Fully Adjusted Models for Serious Illness Conversations (Sics) for All Patients and for High Risk Patients Only

Serious Illness Conversation	Control	Intervention	Adjusted Difference for Intervention Relative to Control, Percentage Points (95% CI)	P Value
**All patient encounters**	1.3% (155/12170)	4.6% (632/13889)	3.3% (2.3-4.5)	<.001
**Fully Adjusted Model**	-	-	3.3% (2.3-4.5)	<.001
**Subgroup analyses**				
Age				
18-35 years	0.4% (3/682)	1.2% (12/982)	-	-
36-50 years	1% (16/1546)	3.2% (64/1998)	-	-
51-64 years	1.5% (63/4130)	4.8% (221/4619)	-	-
≥ 65 years	1.3% (73/5812)	5.3% (335/6290)	-	-
Gender				
Female	1.3% (85/6426)	4.1% (312/7576)	-	-
Male	1.2% (70/5744)	5.1% (320/6313)	-	-
Race/Ethnicity				
White non-Hispanic	1.3% (108/8629)	4.5% (435/9709)	-	-
Black non-Hispanic	1.5% (37/2436)	4.6% (128/2772)	-	-
Other	0.9% (10/1105)	4.9% (69/1408)	-	-
Insurance				
Commercial	1.3% (69/5467)	3.5% (230/6522)	-	-
Medicare	1.3% (80/5953)	5.5% (345/6321)	-	-
Medicaid	0.8% (6/750)	5.4% (57/1046)	-	-
Predicted mortality risk				
0.0 - 0.1	0.3% (26/8247)	1.2% (110/9475)	-	-
>0.1 to 0.2	1.3% (20/1566)	6.8% (108/1596)	-	-
>0.2 to 0.3	2% (13/642)	10.2% (73/713)	-	-
>0.3 to 0.4	3.4% (15/443)	13.4% (65/486)	-	-
>0.4 to 0.5	4.6% (14/303)	18.4% (71/385)	-	-
>0.5	9.2% (62/677)	25.1% (183/730)	-	-
**High-risk patient encounters**	3.6% (77/2125)	15.2% (304/1999)	11.6% (8.2-15.5)	<.001
**Fully Adjusted Model**	-	-	11.6% (8.2-15.5)	<.001
**Subgroup analyses**				
Age				
18-35 years	2.9% (2/70)	12.9% (9/70)	-	-
36-50 years	3.2% (6/189)	13.3% (26/195)	-	-
51-64 years	5% (39/775)	14.9% (104/696)	-	-

≥ 65 years	2.7% (30/1091)	15.9% (165/1038)	-	-
Gender				
Female	3.8% (41/1070)	15% (154/1025)	-	-
Male	3.4% (36/1055)	15.4% (150/974)	-	-
Race/Ethnicity				
White non-Hispanic	3.9% (58/1494)	14.2% (201/1417)	-	-
Black non-Hispanic	3.6% (17/467)	16.9% (69/408)	-	-
Other	1.2% (2/164)	19.5% (34/174)	-	-
Insurance				
Commercial	4.6% (37/809)	11.5% (90/782)	-	-
Medicare	3.3% (38/1149)	16.3% (173/1060)	-	-
Medicaid	1.2% (2/167)	26.1% (41/157)	-	-

© 2020 Manz CR et al. *JAMA Oncology*

eTable 6. Serious Illness Conversations (Sics) for All Patients, Documented Within 7 Days

Serious Illness Conversation	Control	Intervention	Adjusted Difference for Intervention Relative to Control, Percentage Points (95% CI)	P Value
**All patient encounters**	0.9% (114/12170)	3.7% (515/13889)	2.8% (1.8-4)	<.001
**High-risk patient encounters**	2.9% (61/2125)	12.2% (243/1999)	9.3% (6-13.1)	<.001

© 2020 Manz CR et al. *JAMA Oncology*

eTable 7. Serious Illness Conversations (Sics), Adjusted for Clinician Time From SIC Guide Training to Trial Start Date

	Odds Ratio	95% Confidence Interval	P value
SICs, all patients: main model	2.02	1.44-2.83	<.001
Sensitivity model: main model + training interval variable*	2.03	1.44-2.85	<.001
SICs, high risk patients: main model	2.72	1.73-4.28	<.001
Sensitivity model: main model + training interval variable	2.74	1.74-4.31	<.001

* Training interval variable is the clinician-level time from Serious Illness Conversation Guide Training to the start date of the trial

© 2020 Manz CR et al. *JAMA Oncology*

eTable 8. Regression Table for Advance Care Planning Model Including All Patients

Variable	Estimate	P Value	OR (CI)
**Wedge**			
10/7/19 – 11/3/19	0.7919	<.0001	2.21 (1.6-3.04)
7/15/19 – 8/11/19	0.2555	0.0201	1.29 (1.04-1.6)
8/12/19 – 9/8/19	0.6287	<.0001	1.88 (1.41-2.5)
9/9/19 – 10/6/19	0.6313	0.0002	1.88 (1.34-2.63)
**Intervention**			
Pre	Ref	-	Ref
Post	0.3603	0.001	1.43 (1.16-1.78)
**Clinic**			
Lymphoma	Ref	-	Ref
Gastrointestinal	1.5608	<.0001	4.76 (2.23-10.15)
Thoracic	1.5699	0.0001	4.81 (2.17-10.63)
Central Nervous System / Melanoma	0.6904	0.0732	1.99 (0.94-4.24)
Myeloma	0.0772	0.8776	1.08 (0.4-2.89)
Genitourinary	-0.2742	0.5272	0.76 (0.32-1.78)
General oncology	0.431	0.2301	1.54 (0.76-3.11)
Breast	-0.4974	0.3698	0.61 (0.21-1.8)

© 2020 Manz CR et al. *JAMA Oncology*

eTable 9. Regression Table for Advance Care Planning Model Including High Risk Patients Only

Variable	Estimate	P Value	OR (CI)
**Wedge**			
10/7/19 – 11/3/19	0.9919	0.0001	2.7 (1.63-4.46)
7/15/19 – 8/11/19	0.3681	0.0626	1.44 (0.98-2.13)
8/12/19 – 9/8/19	0.7603	0.0033	2.14 (1.29-3.55)
9/9/19 – 10/6/19	0.7732	0.0023	2.17 (1.32-3.57)
**Intervention**			
Pre	Ref	-	Ref
Post	0.6889	<.0001	1.99 (1.43-2.78)
**Clinic**			
Lymphoma	Ref	-	Ref
Gastrointestinal	1.0697	0.0101	2.91 (1.29-6.59)
Thoracic	1.3665	0.0045	3.92 (1.53-10.07)
Central Nervous System / Melanoma	0.4808	0.3259	1.62 (0.62-4.22)
Myeloma	0.1775	0.7052	1.19 (0.48-3)
Genitourinary	0.1164	0.8312	1.12 (0.39-3.28)
General oncology	0.7487	0.1465	2.11 (0.77-5.81)
Breast	-0.3233	0.5029	0.72 (0.28-1.86)

© 2020 Manz CR et al. *JAMA Oncology*

eTable 10. Fully Adjusted Models for Advance Care Planning (Acps) for All Patients and for High Risk Patients Only

Advanced Care Planning	Control	Intervention	Adjusted Difference for Intervention Relative to Control, Percentage Points (95% CI)	P Value
**All patient encounters**	1.9% (231/12170)	4.9% (680/13889)	3% (2.1-4.1)	.001
**Fully Adjusted Model**	-	-	3% (2.1-4.1)	<.001
**Subgroup analyses**				
Age				
18-35 years	0.4% (3/682)	1.3% (13/982)	-	-
36-50 years	1.6% (25/1546)	3.3% (65/1998)	-	-
51-64 years	2.2% (91/4130)	5.1% (237/4619)	-	-
≥ 65 years	1.9% (112/5812)	5.8% (365/6290)	-	-
Gender				
Female	1.9% (120/6426)	4.5% (340/7576)	-	-
Male	1.9% (111/5744)	5.4% (340/6313)	-	-
Race/Ethnicity				
White non-Hispanic	1.9% (162/8629)	4.8% (464/9709)	-	-
Black non-Hispanic	2% (48/2436)	5% (139/2772)	-	-
Other	1.9% (21/1105)	5.5% (77/1408)	-	-
Insurance				
Commercial	1.8% (101/5467)	3.9% (253/6522)	-	-
Medicare	2% (121/5953)	5.8% (369/6321)	-	-
Medicaid	1.2% (9/750)	5.5% (58/1046)	-	-
Predicted mortality risk				
0.0 - 0.1	0.4% (33/8247)	1.2% (118/9475)	-	-
>0.1 to 0.2	2.2% (34/1566)	7.1% (113/1596)	-	-
>0.2 to 0.3	4% (26/642)	11.2% (80/713)	-	-
>0.3 to 0.4	5.4% (24/443)	14% (68/486)	-	-
>0.4 to 0.5	6.6% (20/303)	20.5% (79/385)	-	-
>0.5	13.1% (89/677)	27.4% (200/730)	-	-
**High-risk patient encounters**	5.8% (124/2125)	17.5% (350/1999)	11.7% (8.4-15.7)	<.001
**Fully Adjusted Model**	-	-	11.7% (8.4-15.7)	<.001
**Subgroup analyses**				
Age				
18-35 years	2.9% (2/70)	12.9% (9/70)	-	-
36-50 years	6.3% (12/189)	15.4% (30/195)	-	-
51-64 years	7% (54/775)	17% (118/696)	-	-

© 2020 Manz CR et al. *JAMA Oncology*

≥ 65 years	5.1% (56/1091)	18.6% (193/1038)	-	-
Gender				
Female	5.9% (63/1070)	17.5% (179/1025)	-	-
Male	5.8% (61/1055)	17.6% (171/974)	-	-
Race/Ethnicity				
White non-Hispanic	6.2% (93/1494)	16.3% (231/1417)	-	-
Black non-Hispanic	4.7% (22/467)	19.6% (80/408)	-	-
Other	5.5% (9/164)	22.4% (39/174)	-	-
Insurance				
Commercial	6.7% (54/809)	14.3% (112/782)	-	-
Medicare	5.7% (66/1149)	18.5% (196/1060)	-	-
Medicaid	2.4% (4/167)	26.8% (42/157)	-	-

© 2020 Manz CR et al. *JAMA Oncology*

eTable 11. Adjusted Change in Advance Care Planning Conversations (Sics), Documented Within 7 days

Advanced Care Planning	Control	Intervention	Adjusted Difference for Intervention Relative to Control, Percentage Points (95% CI)	P Value
**All patient encounters**	1% (120/12170)	3.7% (515/13889)	2.7% (1.7-3.9)	0.0001
**High-risk patient encounters**	3.1% (66/2125)	12.4% (247/1999)	9.3% (6-13.1)	<.0001

© 2020 Manz CR et al. *JAMA Oncology*

eTable 12. Adjusted Change in Serious Illness Conversations (Sics) and Advanced Care Planning for Non-High-Risk Patients

Outcome	Control	Intervention	Adjusted Difference for Intervention Relative to Control, Percentage Points (95% CI) [a]	P Value
**Non high-risk patient encounters: Serious Illness Conversations**	0.7% (76/10298)	2.5% (300/12130)	1.8% (1.0-2.7)	0.0524
**Non high-risk patient encounters: Advance Care Planning**	1.1% (117/10298)	2.8% (339/12130)	1.7% (0.9-2.5)	0.35

[a] This post-hoc analysis was performed using the same model used in the primary and secondary outcomes reported in the manuscript.

© 2020 Manz CR et al. *JAMA Oncology*
