# Supplementary material for: Effect of Integrating Machine Learning Mortality Estimates With Behavioral Nudges to Clinicians on Serious Illness Conversations Among Patients With Cancer: A Stepped-Wedge Cluster Randomized Clinical Trial
Source: JAMA Oncol. 2020 Oct 15;6(12):e204759. doi: 10.1001/jamaoncol.2020.4759 (PMC7563672; doi:10.1001/jamaoncol.2020.4759)
Supplement: Supplement 3. — Data Sharing Statement [file jamaoncol-e204759-s003.pdf]

Data Sharing Statement

Manz. Effect of Integrating Machine Learning Mortality Estimates With Behavioral Nudges to Clinicians on Serious Illness Conversations Among Patients With Cancer. *JAMA Oncol*. Published October 15, 2020. 10.1001/jamaoncol.2020.4759

Data
Data available: No

Additional Information
Explanation for why data not available: The study involves extensive electronic medical record data for all trial participants that cannot be accessed outside of the health system.
